# Diagnostic Performance of Blood-Based Inflammatory Indices, Including a Novel Composite Score, for Estimating Fecal Calprotectin Levels in Pediatric Inflammatory Bowel Disease

**DOI:** 10.3390/jcm15083046

**Published:** 2026-04-16

**Authors:** Abdulkerim Elmas, Mustafa Akçam

**Affiliations:** Division of Pediatric Gastroenterology, Hepatology and Nutrition, Department of Pediatrics, Faculty of Medicine, Suleyman Demirel University, 32260 Isparta, Türkiye; makcam32@gmail.com

**Keywords:** fecal calprotectin, pediatric IBD, inflammatory indices, NLR, SII, gastrointestinal inflammation index, noninvasive biomarkers

## Abstract

**Objectives:** To evaluate the association between fecal calprotectin (FC) levels and routinely available blood-based inflammatory indices measured during the same clinical episode in pediatric patients, as well as to assess the diagnostic performance of a novel composite parameter, the Gastrointestinal Inflammation Index (GII). **Methods:** This retrospective cross-sectional study included pediatric patients who underwent simultaneous testing for FC, complete blood count, C-reactive protein, and albumin between 2022 and 2025. Hematological inflammatory indices, including neutrophil-to-lymphocyte ratio (NLR), platelet-to-lymphocyte ratio (PLR), red cell distribution width (RDW), platelet mass index (PMI), systemic immune-inflammation index (SII), and the newly developed GII, were calculated. Correlations between FC and inflammatory indices were analyzed. Receiver operating characteristic (ROC) curve analysis was performed to evaluate diagnostic performance, and multivariate logistic regression was used to identify independent predictors of FC positivity. **Results:** Elevated FC levels were significantly associated with higher C-reactive protein levels, lower albumin concentrations, and increased values of RDW, PMI, SII, and GII (all *p* < 0.001). GII scores increased progressively across FC categories. In ROC analysis, GII demonstrated the highest discriminatory ability for predicting FC positivity (AUC = 0.660), followed by SII and PMI. In multivariate logistic regression analysis, only NLR remained an independent predictor of FC positivity (OR = 0.65, 95% CI: 0.44–0.97; *p* = 0.033). **Conclusions:** Blood-based inflammatory indices show significant associations with fecal calprotectin levels in pediatric inflammatory bowel disease. The novel GII may reflect the integrated systemic inflammatory burden related to intestinal involvement, while NLR appears to be a robust and practical independent marker. These indices may serve as adjunctive, rapid, and cost-effective supportive tools in clinical decision-making, although their moderate diagnostic performance limits their use as standalone screening markers.

## 1. Introduction

Inflammatory bowel disease (IBD) is a chronic condition that requires close monitoring and repeated assessments of disease activity due to its increasing incidence in childhood and its heterogeneous clinical course.

Clinical scoring systems, such as the Pediatric Ulcerative Colitis Activity Index (PUCAI) and the Pediatric Crohn’s Disease Activity Index (PCDAI), form the basis of standardized follow-up in pediatric ulcerative colitis (UC) and Crohn’s disease (CD). However, they may not always adequately reflect mucosal disease activity. Endoscopic imaging techniques, on the other hand, are invasive and therefore not feasible for frequent use in children. In this context, fecal calprotectin (FC) is recognized as an important biomarker that can non-invasively and sensitively indicate intestinal inflammation [1,2]. In terms of diagnostic thresholds, FC levels below 50 µg/g are generally considered normal in adults and children aged ≥4 years; levels between 50 and 150/200 µg/g are regarded as borderline, while values above 250 µg/g have been reported to be more strongly associated with active mucosal disease. However, it has also been noted that reference ranges may vary with age, and physiologically higher FC levels may be observed in younger children [3,4].

In recent years, the STRIDE-II and Porto consensus statements have placed FC at the center of treat-to-target monitoring in pediatric IBD [5]. In terms of correlation with endoscopic activity, FC levels have demonstrated significant associations in both UC and CD, with multicenter studies and systematic reviews showing that levels particularly around 250 µg/g can support clinical decision-making regarding endoscopic remission or activity. In pediatric cohorts, the correlation between FC and PUCAI/PCDAI has been reported to be of moderate strength [6,7].

Due to its ability to reflect the presence of inflammation and endoscopic activity, FC is recommended in clinical guidelines as part of treat-to-target monitoring. However, limited laboratory infrastructure and test-related costs hinder its widespread use and accessibility in many centers. These delays often compel clinicians to rely on alternative biomarkers at the point of decision-making [8,9,10].

Blood-based inflammatory indices are derived from components such as the neutrophil-to-lymphocyte ratio (NLR), platelet-to-lymphocyte ratio (PLR), systemic immune-inflammation index (SII = neutrophil × platelet/lymphocyte), and platelet mass index (PMI = platelet count × mean platelet volume [MPV]). In adult UC, increasing evidence suggests that NLR and PLR are associated with disease activity, while SII may demonstrate superior performance in predicting remission and relapse compared to NLR and PLR. MPV has been reported to decrease during active UC, whereas platelet count may be associated with disease activity and relapse risk [11,12,13,14,15]. These markers also provide a basis for hypothesis generation in the pediatric population; however, studies systematically evaluating the concurrent correlation and predictive power of blood-based composite indices with FC in children are limited. On the other hand, basic hematological parameters such as red cell distribution width (RDW) have also been suggested to be associated with IBD activity and may show significant elevation during active disease phases [16]. However, comprehensive pediatric studies evaluating the combined correlation and predictive value of these biomarkers with FC remain limited.

In this study, we aimed to evaluate the relationship between inflammatory parameters such as NLR, PLR, PMI, SII, and RDW and concurrent FC levels in pediatric UC and CD cases. FC was analyzed both categorically (negative/borderline/positive) and within positive subgroups (150–499, 500–999, and ≥1000 µg/g) to assess its discriminatory capacity. Additionally, a novel ‘Gastrointestinal Inflammation Index (GII)’ derived from these markers was proposed, and its potential applicability in clinical decision-making was explored.

## 2. Methods

### 2.1. Study Design and Study Population

This single-center, retrospective cross-sectional study was conducted at our clinic between 2022 and 2025. Pediatric patients aged 0–18 years who had simultaneous measurements of FC, complete blood count (CBC), C-reactive protein (CRP), and albumin during the same clinical episode were included. Demographic, hematological, and biochemical data were retrospectively obtained from the electronic hospital database. Patients with missing data or non-simultaneous sampling were excluded from the study.

### 2.2. Laboratory Measurements and Variable Definitions

FC was quantitatively analyzed using the Euroimmun AG^®^ Calprotectin ELISA kit (Euroimmun AG GmbH, Lübeck, Germany). Samples were stored at 2–8 °C in accordance with the manufacturer’s recommendations and analyzed within 72 h. Complete blood count parameters were measured using the DxH 900 Hematology Analyzer (Beckman Coulter Diagnostics, Nyon, Switzerland).

Inflammatory markers based on complete blood count were calculated. Accordingly, NLR was defined as NEU/LYM, PLR as PLT/LYM, PMI as PLT × MPV, SII as PLT × NEU/LYM, and RDW was analyzed using the standard formula.

FC was first analyzed as a continuous variable to assess its correlation with blood-based biomarkers. To reflect clinical decision-making processes, FC levels were also classified categorically: negative (<50 µg/g), borderline (50–149 µg/g), and positive (≥150 µg/g). To further analyze the severity of inflammation within the positive group, a secondary subclassification was applied: Positive-1 (150–499 µg/g), Positive-2 (500–999 µg/g), and Positive-3 (≥1000 µg/g). All analyses were conducted using CBC and biochemical data obtained from the same clinical episode as the FC measurement.

The study aimed to develop a clinically applicable composite score based on blood-derived inflammatory parameters. This led to the creation of the GII. Each parameter was scored between 0 and 2 points according to the threshold values presented in Table 1, and a total GII score was calculated. Accordingly, a score of 0 was considered low risk, 1–2 points as a gray zone, 3 points as at-risk, and ≥4 points as high risk. This scoring system was applied to all cases with available FC results.

### 2.3. Statistical Analysis

Statistical analyses were performed using IBM SPSS Statistics v27 (IBM Corp., Armonk, NY, USA). The distribution of continuous variables was assessed using the Shapiro–Wilk test. Non-normally distributed data were presented as median (IQR), while normally distributed variables were expressed as mean ± standard deviation (SD). Group differences were analyzed as follows: categorical variables with the Chi-square (χ^2^) test; non-normally distributed continuous variables with the Kruskal–Wallis H test and, when necessary, the Mann–Whitney U test with Bonferroni correction; and normally distributed continuous variables with one-way ANOVA. Correlation analyses were performed using Spearman’s rho test. A *p*-value < 0.05 was considered statistically significant.

### 2.4. Ethical Approval

Approved by the Ethics Committee of Süleyman Demirel University Faculty of Medicine (Approval No: 2025/38, Date: 20 September 2025). The study was conducted in accordance with the principles of the Declaration of Helsinki.

## 3. Results

A total of 265 pediatric patients with 376 samples, in which FC and blood tests (complete blood count, CRP, and albumin) were evaluated during the same clinical episode, were included in the study. Of the cases, 51.6% (*n* = 194) were male, and the median age was 11 years (IQR: 7–15; range: 1–17 years). Among all samples, 31 patients (11.6%) were diagnosed with IBD. FC levels were found to be significantly higher in children with IBD (median: 796.3 µg/g, IQR: 130.2–1112.0), and this difference was statistically significant compared to the non-IBD group (median: 24.9 µg/g, IQR: 7.4–89.5; *p* < 0.001).

Cases were divided into three groups based on FC levels: negative (<50 µg/g, *n* = 217), borderline (50–149 µg/g, *n* = 52), and positive (≥150 µg/g, *n* = 107). There were no significant differences between the groups in terms of age (*p* = 0.24) or sex distribution (*p* = 0.61). Analysis of laboratory parameters demonstrated a more pronounced inflammatory response in the positive FC group. In this group, CRP levels were significantly higher (*p* < 0.001), while albumin levels were significantly lower (*p* = 0.003). Additionally, neutrophil and platelet counts were significantly elevated in the positive group (*p* = 0.016 and *p* < 0.001, respectively).

Hemoglobin levels differed significantly among the groups (*p* = 0.023), and RDW was significantly higher in the positive group (*p* < 0.001). Blood-based inflammatory indices—NLR, PLR, PMI, and SII—showed a progressive increase in parallel with FC levels, with statistically significant differences observed between the groups (*p* = 0.004, *p* < 0.001, *p* < 0.001, and *p* < 0.001, respectively). GII scores also differed significantly across FC groups (*p* < 0.001). The difference between the negative and positive FC groups was statistically significant (*p* < 0.001), whereas no significant differences were found between the borderline group and the other two groups (negative–borderline: *p* = 0.17; borderline–positive: *p* = 0.14) (Table 2).

The 107 cases in the positive FC group were subdivided into three groups based on FC levels: Positive-1 (150–499 µg/g), Positive-2 (500–999 µg/g), and Positive-3 (≥1000 µg/g). There were no significant differences among these groups in terms of age, sex, or basic hematological parameters. However, the GII score increased progressively in parallel with FC levels, and this increase was statistically significant (*p* = 0.02). Multiple comparisons using the Mann–Whitney U test revealed that the differences were between the Positive-1 and Positive-2 groups (*p* = 0.019) and between the Positive-1 and Positive-3 groups (*p* = 0.030). These findings indicate that as FC levels increase, the GII score also rises gradually, suggesting that components of systemic inflammation correlate with the severity of intestinal inflammation (Table 3).

**Figure 1:**
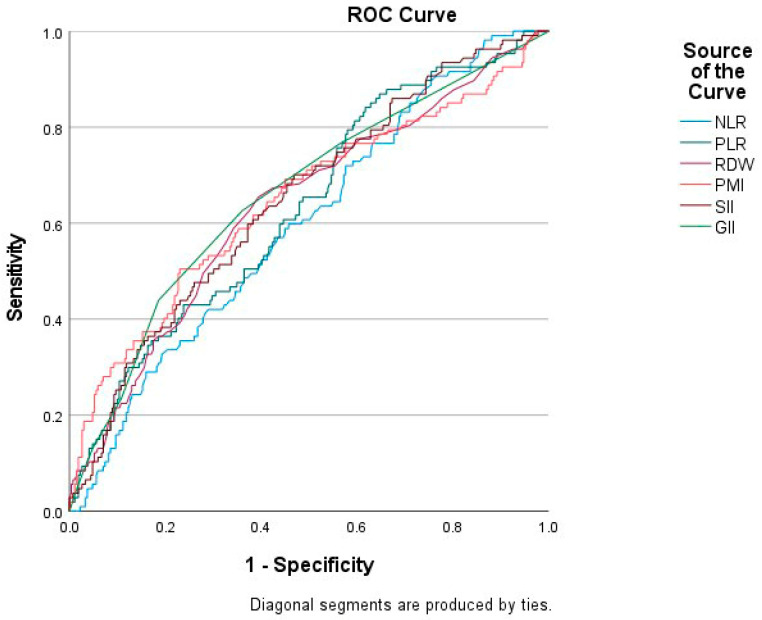
ROC Curves for Blood-Based Indices Predicting Fecal Calprotectin Positivity.

The diagnostic performance in predicting FC positivity was evaluated using ROC analysis. All hematological indices (NLR, PLR, RDW, PMI, SII, and GII) demonstrated significant discriminatory power (*p* < 0.01). The AUC values were as follows: NLR: 0.597, PLR: 0.632, RDW: 0.635, PMI: 0.646, SII: 0.648, and GII: 0.660. Among these, GII showed the highest discriminatory ability. SII and PMI also demonstrated comparable levels of diagnostic performance (Figure 1).

In the multivariate logistic regression analysis, the overall model was found to be significant (χ^2^ = 51.94, df = 6, *p* < 0.001) and explained 18.5% of the variance (Nagelkerke R^2^ = 0.185). The Hosmer–Lemeshow test (*p* = 0.461) confirmed a good model fit.

Among the variables analyzed, only NLR was identified as an independent predictor with statistical significance (OR = 0.65, 95% CI: 0.44–0.97, *p* = 0.033). RDW (*p* = 0.070) and SII (*p* = 0.077) were close to significance, while GII, PLR, and PMI did not demonstrate independent predictive value within the regression model. These findings suggest that although hematological indices are associated with FC levels, only NLR remained a significant independent marker when multiple variables were considered together.

## 4. Discussion

In this study, hematological parameters measured during the same clinical episode as FC were analyzed to assess the relationship between systemic inflammation markers and intestinal inflammation in children. The findings demonstrated that blood-based biomarkers reflecting systemic inflammation were significantly associated with FC levels, and some parameters exhibited statistically significant diagnostic performance in predicting fecal inflammation. In our study, the strongest correlation and highest ROC performance were observed with the newly developed GII. The integration of various hematological components—such as neutrophils, lymphocytes, platelets, and RDW—within this index suggests that it may better capture the multidimensional nature of systemic inflammation. However, the overall discriminatory performance of these indices remained modest (AUC values around 0.60–0.66), indicating that they should be interpreted cautiously and not be used as standalone diagnostic tools.

FC is a widely used and reliable marker of mucosal activity in both the diagnosis and monitoring of IBD [17]. However, the cost of the test, the requirement for laboratory infrastructure, and delays in obtaining results increase the need for alternative markers, particularly in pediatric clinical settings. The hematological indices analyzed in this study hold potential as rapid, low-cost, and easily accessible alternative biomarkers in clinical practice.

In the literature, hematological parameters have been reported to be associated with FC and disease activity. Plume et al. stated that, in pediatric IBD cases, FC levels parallel clinical activity scores and correlate with certain hematological parameters [17]. These findings support the significance of the positive correlations observed in our study.

Studies investigating the diagnostic value of hematological indices have primarily focused on NLR and SII. In pediatric IBD research, there is growing evidence supporting the utility of NLR in both diagnosis and assessment of disease activity, which aligns with our finding that NLR remained an independent predictor in the multivariate model [18,19,20]. In adult populations, SII has been reported to be associated with endoscopic activity and even mucosal and histological healing, which provides a rationale for its evaluation in the pediatric population [21]. On the other hand, evidence linking RDW with IBD activity has been available for quite some time. As an inexpensive and accessible ancillary marker, the relatively modest and variable associations observed in our findings suggest that RDW may be considered, particularly for use as a screening tool [16,22]. In a study conducted on adult IBD patients, SII was reported to show a strong correlation with endoscopic activity and achieved a high diagnostic accuracy in ROC analysis, with an AUC of 0.86 [21]. Additionally, Kawamoto et al. demonstrated that NLR could be used for the non-invasive assessment of CD activity [23]. In our study, the highest AUC value was obtained with GII, followed by SII and PMI. This finding suggests that GII, in particular, may more comprehensively reflect the systemic effects of the inflammatory process. However, in the multivariate regression analysis, only NLR demonstrated statistical significance as an independent predictor of fecal inflammation. This indicates that NLR may serve as a strong parameter reflecting IBD activity in children.

When interpreted alongside previous literature, our findings suggest that, the highest AUC value in our study was obtained with GII, followed by SII and PMI. This finding suggests that GII, in particular, may provide a more comprehensive reflection of the systemic impact of the inflammatory process. In the literature, SII has been reported to offer high diagnostic accuracy in both the diagnosis and activity assessment of UC. For example, in the study by Yan et al., SII demonstrated high specificity and sensitivity in distinguishing patients with UC from healthy individuals, with an AUC of 0.861. In the same study, SII was also shown to correlate significantly with clinical activity (Mayo score), endoscopic activity (MES), and histological inflammation (Nancy score) [21]. Similarly, in the study by Xie et al., SII was found to be significantly elevated in patients with active UC and achieved an AUC of 0.711 in ROC analysis [24]. Lin et al. reported that SII provided higher diagnostic accuracy than PLR and NLR as a non-invasive indicator in active disease [25]. In a more recent study, Yan J et al. reported that SII outperformed other indices in predicting both clinical remission and relapse risk in UC patients receiving vedolizumab therapy, with an AUC of 0.793 [12]. These findings support the positive association observed between SII and FC in our study and further strengthen the potential utility of SII in the pediatric IBD population.

These findings suggest that the indices are not sufficient as standalone tools. However, they may still provide valuable guidance in clinical decision-making. In cases where GII or SII values are markedly elevated, it may be appropriate for the clinician to consider the likelihood of intestinal inflammation and proceed with FC testing or further diagnostic evaluation as needed. Thus, these indices may function as supportive indicators in the early stages of clinical decision-making.

One of the strengths of our study is its large pediatric sample in which fecal and hematological parameters were evaluated simultaneously. First, the fact that all indices were derived from routine laboratory data demonstrates the practical applicability of the method. However, the retrospective and single-center design may limit the generalizability of the findings. In addition, the relatively small number of patients diagnosed with IBD (*n* = 31) restricts the strength of IBD-specific subgroup analyses. The study population also included a heterogeneous group of patients, which may have influenced the observed associations. Moreover, the absence of clinical activity scores such as PUCAI and PCDAI in the analysis prevents a full assessment of the relationship between biomarker levels and the symptomatic expression of inflammation. The GII scoring system represents a novel composite parameter; however, its threshold values were not derived from data-driven optimization methods such as ROC-based cutoff analysis, which may limit its precision and reproducibility. Furthermore, the absence of an external validation cohort limits the generalizability and clinical applicability of the proposed scoring system. In addition, the absence of concurrent clinical activity indices and endoscopic scoring limits the ability to directly correlate hematological markers with mucosal healing or disease severity. Another limitation is the lack of detailed information on medication use, including corticosteroids, immunomodulators, and biologic therapies, which may have affected hematological parameters. Additionally, subgroup analyses according to disease subtype (ulcerative colitis vs. Crohn’s disease) were not performed, despite known differences in inflammatory profiles. Furthermore, the lack of significant differentiation involving the borderline FC group suggests limited discriminative capacity in intermediate clinical scenarios. Finally, the relatively low proportion of IBD cases in the cohort may restrict the generalizability of the findings to populations with a higher pretest probability of inflammatory bowel disease.

## 5. Conclusions

In conclusion, this study demonstrated significant associations between FC levels and hematological markers of inflammation. Composite indices such as GII may effectively reflect the systemic manifestations of intestinal inflammation and offer a rapid, low-cost adjunct to clinical decision-making. The use of such non-invasive markers may be particularly valuable in the clinical management of pediatric patients. Future prospective, multicenter studies are needed to more comprehensively evaluate the diagnostic and monitoring performance of these indices.

## Figures and Tables

**Table 1:** Gastrointestinal Inflammation Index (GII) Scoring System.

Parameter	0 Point	1 Point	2 Points
PLR	<150	150–249	≥250
RDW	<14.5	14.5–15.9	≥16.0
SII	<700	700–1199	≥1200

Abbreviations: PLR: Platelet-to-Lymphocyte Ratio, RDW: Red Cell Distribution Width, SII: Systemic Immune-Inflammation Index.

**Table 2:** Comparison of Demographic Characteristics, Laboratory Parameters, and Inflammation Indices According to Fecal Calprotectin Levels.

Variable	Negative *n* = 217	Borderline *n* = 52	Positive *n* = 107	*p* *
Sex, male, *n* (%)	116 (53.5)	24 (46.2)	21 (43.8)	0.61
Age, year, median (IQR)	12 (7–16)	10 (7–15)	13 (8–15)	0.24
CRP, mg/L, median (IQR)	0.7 (0.2–2.0)	0.6 (0.3–6.6)	1.5 (0.4–5.3)	**<0.001**
Albumin, g/L, median (IQR)	4.52 (4.4–4.8)	4.50 (4.30–4.77)	4.5 (4.4–4.7)	**0.003**
WBC, 10^3^/µL, median (IQR)	7.1 (6.0–9.5)	7.7 (6.0–9.8)	6.8 (5.3–9.6)	0.41
Neutrophil, 10^3^/µL, median (IQR)	3.8 (2.8–5.1)	4.25 (2.9–5.7)	3.6 (2.9–6.1)	**0.016**
Lymphocyte, 10^3^/µL, median (IQR)	2.3 (1.8–3.1)	2.1 (1.7–2.8)	2.2 (1.5–2.7)	0.31
Platelet, 10^3^/µL, median (IQR)	282 (246–346)	294 (245.5–375.2)	327 (247.7–415.7)	**<0.001**
MPV, fL, mean (±SD)	8.1 (0.9)	7.8 (0.9)	8.0 (0.8)	0.56 **
Hb, g/dL, median (IQR)	13.8 (12.8–14.7)	13.2 (12.2–14.4)	13.5 (12.8–14.2)	**0.023**
RDW, %, median (IQR)	14.0 (13.5–15.0)	14.2 (13.3–15.3)	14.9 (13.6–16.1)	**<0.001**
NLR, median (IQR)	1.63 (1.0–2.4)	1.98 (1.25–2.67)	2 (1.1–2.4)	**0.004**
PLR, median (IQR)	125.2 (93.1–156.3)	135.1 (100.5–179.3)	142.1 (118.7–208.6)	**<0.001**
PMI, median (IQR)	2366.0 (2031.0–2679.0)	2320.5 (2048.5–2824.0)	2590.5 (2068.25–3150.25)	**<0.001**
SII, median (IQR)	444.0 (296.0–699.5)	555.5 (338.0–812.5)	589 (422.7–899.25)	**<0.001**
GII score, median (IQR) min–max	1.0 (0–2.0)	1.0 (0–3.0)	2 (1.0–3.0)	**<0.001**
Negative–Borderline				0.17 ^¥^
Negative–Positive				**<0.001** ^¥^
Borderline–Positive				0.14 ^¥^

* The Chi-square test was used for categorical variables, and the Kruskal–Wallis H test was used for numerical variables; ** One-way ANOVA was used; ^¥^ Mann–Whitney U was used. **Abbreviations**: CRP: C-reactive protein; Hb: Hemoglobin; NLR: Neutrophil-to-Lymphocyte Ratio; PLR: Platelet-to-Lymphocyte Ratio; PMI: Platelet Mass Index; SII: Systemic Immune-Inflammation Index; RDW: Red Cell Distribution Width; MPV: Mean Platelet Volume; SD: Standard Deviation; GII: Gastrointestinal Inflammation Index. Bold values indicate statistically significant results (*p* < 0.05).

**Table 3:** Comparison of Laboratory Parameters and Inflammation Indices Among Positive Fecal Calprotectin Subgroups (150–499, 500–999, and ≥1000 µg/g).

Variable	Positive-1 *n* = 48	Positive-2 *n* = 30	Positive-3 *n* = 29	*p* *
Sex, male, *n* (%)	21 (43.8)	14 (46.7)	19 (65.5)	0.16
Age, year, median (IQR)	11 (7–15)	14 (8–15)	14 (11–18)	0.07
CRP, mg/L, median (IQR)	7.22 (0.4–5.5)	1.1 (0.6–8.9)	4.3 (0.3–12.2)	0.67
Albumin, g/L, median (IQR)	4.42 (4.3–4.7)	4.3 (3.9–4.5)	4.5 (4.0–4.7)	0.13
WBC, 10^3^/µL, median (IQR)	6.9 (5.3–9.8)	8.4 (6.6–14.0)	8.3 (6.4–11.4)	0.06
Neutrophil, 10^3^/µL, median (IQR)	3.6 (2.8–6.0)	5.4 (3.5–8.6)	5.0 (3.3–6.1)	0.07
Lymphocyte, 10^3^/µL, median (IQR)	2.3 (1.5–2.8)	2.1 (1.7–2.9)	2.2 (1.7–2.6)	0.92
Platelet, 10^3^/µL, median (IQR)	328.5 (247–407)	341.5 (284–500)	333 (288–493)	0.33
MPV, fL, mean (±SD)	8.1 (0.9)	8.1 (0.79)	8.01 (1.04)	0.87 **
Hb, g/dL, median (IQR)	13.5 (12.7–14.2)	12.8 (11.2–14.0)	13.7 (12.3–14.5)	0.11
RDW, %, median (IQR)	14.5 (13.5–15.2)	14.8 (14.2–19.1)	15.5 (13.8–17.7)	0.10
NLR, median (IQR)	2.0 (1.1–2.5)	2.3 (1.6–3.6)	2.1 (1.2–3.3)	0.21
PLR, median (IQR)	136.8 (115.2–208.6)	144.1 (114.5–227.5)	164.7 (115–205)	0.65
PMI, median (IQR)	2601.0 (2068.2–3130.5)	2796 (2410.7–3928.5)	2864 (2310.5–3832.0)	0.20
SII, median (IQR)	589.0 (402.5–852.0)	880 (474.7–1526.5)	766 (361–1394)	0.058
GII score, median (IQR)	1.0 (0–3.0)	3.0 (1.0–4.0)	3 (1.5–4)	**0.02**
Positive-1/Positive-2				**0.019** ^¥^
Positive-1/Positive-3				**0.030** ^¥^
Positive-2/Positive-3				0.799 ^¥^

* The Chi-square test was used for categorical variables, and the Kruskal–Wallis H test was used for numerical variables; ** One-way ANOVA was used; ^¥^ Mann–Whitney U was used. Abbreviations: CRP: C-reactive protein; Hb: Hemoglobin; NLR: Neutrophil-to-Lymphocyte Ratio; PLR: Platelet-to-Lymphocyte Ratio; PMI: Platelet Mass Index; SII: Systemic Immune-Inflammation Index; RDW: Red Cell Distribution Width; MPV: Mean Platelet Volume; SD: Standard Deviation; GII: Gastrointestinal Inflammation Index. Bold values indicate statistically significant results (*p* < 0.05).

## Data Availability

The data presented in this study are not publicly available due to privacy and ethical restrictions.

## References

[1] Turner D., Hyams J., Markowitz J., Lerer T., Mack D.R., Evans J., Pfefferkorn M., Rosh J., Kay M., Crandall W. (2009). Appraisal of the pediatric ulcerative colitis activity index (PUCAI). Inflamm. Bowel Dis..

[2] Hyams J., Markowitz J., Otley A., Rosh J., Mack D., Bousvaros A., Kugathasan S., Pfefferkorn M., Tolia V., Evans J. (2005). Evaluation of the pediatric crohn disease activity index: A prospective multicenter experience. J. Pediatr. Gastroenterol. Nutr..

[3] Dhaliwal A., Zeino Z., Tomkins C., Cheung M., Nwokolo C., Smith S., Harmston C., Arasaradnam R.P. (2015). Utility of faecal calprotectin in inflammatory bowel disease (IBD): What cut-offs should we apply?. Frontline Gastroenterol..

[4] Oord T., Hornung N. (2014). Fecal calprotectin in healthy children. Scand. J. Clin. Lab. Investig..

[5] Turner D., Ricciuto A., Lewis A., D’Amico F., Dhaliwal J., Griffiths A.M., Bettenworth D., Sandborn W.J., Sands B.E., Reinisch W. (2021). STRIDE-II: An Update on the Selecting Therapeutic Targets in Inflammatory Bowel Disease (STRIDE) Initiative of the International Organization for the Study of IBD (IOIBD): Determining Therapeutic Goals for Treat-to-Target strategies in IBD. Gastroenterology.

[6] D’Haens G., Ferrante M., Vermeire S., Baert F., Noman M., Moortgat L., Geens P., Iwens D., Aerden I., Van Assche G. (2012). Fecal calprotectin is a surrogate marker for endoscopic lesions in inflammatory bowel disease. Inflamm. Bowel Dis..

[7] Kapel N., Ouni H., Benahmed N.A., Barbot-Trystram L. (2023). Fecal Calprotectin for the Diagnosis and Management of Inflammatory Bowel Diseases. Clin. Transl. Gastroenterol..

[8] Turner D., Ruemmele F.M., Orlanski-Meyer E., Griffiths A.M., de Carpi J.M., Bronsky J., Veres G., Aloi M., Strisciuglio C., Braegger C.P. (2018). Management of Paediatric Ulcerative Colitis, Part 1: Ambulatory Care-An Evidence-based Guideline from European Crohn’s and Colitis Organization and European Society of Paediatric Gastroenterology, Hepatology and Nutrition. J. Pediatr. Gastroenterol. Nutr..

[9] Turner D., Ruemmele F.M., Orlanski-Meyer E., Griffiths A.M., de Carpi J.M., Bronsky J., Veres G., Aloi M., Strisciuglio C., Braegger C.P. (2018). Management of Paediatric Ulcerative Colitis, Part 2: Acute Severe Colitis-An Evidence-based Consensus Guideline from the European Crohn’s and Colitis Organization and the European Society of Paediatric Gastroenterology, Hepatology and Nutrition. J. Pediatr. Gastroenterol. Nutr..

[10] Hejl J., Theede K., Møllgren B., Madsen K.V., Heidari A., Steig A.Á., Fenger M. (2018). Point of care testing of fecal calprotectin as a substitute for routine laboratory analysis. Pract. Lab. Med..

[11] Zhang M.H., Wang H., Wang H.G., Wen X., Yang X.Z. (2021). Effective immune-inflammation index for ulcerative colitis and activity assessments. World J. Clin. Cases.

[12] Yan J., Wu J., Wang R., Meng P., Liu A., Xu Y. (2025). The systemic immune-inflammation index is superior to predicting clinical remission and relapse for ulcerative colitis patients treated with vedolizumab. Front. Med..

[13] Yüksel O., Helvaci K., Başar O., Köklü S., Caner S., Helvaci N., Abayli E., Altiparmak E. (2009). An overlooked indicator of disease activity in ulcerative colitis: Mean platelet volume. Platelets.

[14] Chen Z., Lu Y., Wu J., Zhang H. (2021). Clinical significance of blood platelets and mean platelet volume in patients with ulcerative colitis. J. Int. Med. Res..

[15] Nakarai A., Kato J., Hiraoka S., Takashima S., Inokuchi T., Takahara M., Sugihara Y., Harada K., Okada H. (2018). An Elevated Platelet Count Increases the Risk of Relapse in Ulcerative Colitis Patients with Mucosal Healing. Gut Liver.

[16] Song C.S., Park D.I., Yoon M.Y., Seok H.S., Park J.H., Kim H.J., Cho Y.K., Sohn C.I., Jeon W.K., Kim B.I. (2012). Association between red cell distribution width and disease activity in patients with inflammatory bowel disease. Dig. Dis. Sci..

[17] Plume J.L., De A., Mutalib M. (2024). Assessing the correlation between fecal calprotectin, blood markers and disease activity in pediatric inflammatory bowel disease. Ann. Gastroenterol..

[18] Metwally R.H. (2022). Can Neutrophil/Lymphocyte Ratio Assess Inflammatory Bowel Disease Activity and Severity in Children?. Turk. J. Gastroenterol. Off. J. Turk. Soc. Gastroenterol..

[19] Aziz B., Belaghi R., Huynh H., Jacobson K., Mack D.R., Deslandres C., Otley A., DeBruyn J., El-Matary W., Crowley E. (2025). Neutrophil-to-Lymphocyte Ratio at Diagnosis Predicts Colonoscopic Activity in Pediatric Inflammatory Bowel Diseases. Clin. Transl. Gastroenterol..

[20] Şimşek-Onat P., Hizarcioglu-Gulsen H., Ergen Y.M., Gumus E., Özen H., Demir H., Özen S., Saltık-Temizel İ.N. (2023). Neutrophil-to-Lymphocyte Ratio: An Easy Marker for the Diagnosis and Monitoring of Inflammatory Bowel Disease in Children. Dig. Dis. Sci..

[21] Yan J., Deng F., Tan Y., Zhou B., Liu D. (2023). Systemic immune-inflammation index as a potential biomarker to monitor ulcerative colitis. Curr. Med. Res. Opin..

[22] Katsaros M., Paschos P., Giouleme O. (2020). Red cell distribution width as a marker of activity in inflammatory bowel disease: A narrative review. Ann. Gastroenterol..

[23] Kawamoto M., Higashi D., Kinjo K., Takatsu N., Miyasaka Y., Arima H., Nimura S., Hisabe T., Watanabe M. (2025). Neutrophil-to-Lymphocyte Ratio as a Biomarker for Postoperative Complications in Crohn’s Disease. In Vivo.

[24] Xie Y., Zhuang T., Ping Y., Zhang Y., Wang X., Yu P., Duan X. (2021). Elevated systemic immune inflammation index level is associated with disease activity in ulcerative colitis patients. Clin. Chim. Acta Int. J. Clin. Chem..

[25] Lin H., Bai Z., Wu Q., Chu G., Zhang Y., Guo X., Qi X. (2022). Inflammatory Indexes for Assessing the Severity and Disease Progression of Ulcerative Colitis: A Single-Center Retrospective Study. Front. Public Health.

